# High-Throughput Sequencing Enhanced Phage Display Identifies Peptides That Bind Mycobacteria

**DOI:** 10.1371/journal.pone.0077844

**Published:** 2013-11-12

**Authors:** Nqobile A. C. Ngubane, Lionel Gresh, Thomas R. Ioerger, James C. Sacchettini, Yanjia J. Zhang, Eric J. Rubin, Alexander Pym, Makobetsa Khati

**Affiliations:** 1 Emerging Health Technologies Platform, Council for Scientific and Industrial Research, Biosciences Unit, Pretoria, Gauteng, South Africa; 2 KwaZulu-Natal Research Institute for Tuberculosis and Human Immunodeficiency Virus, Nelson R. Mandela School of Medicine, University of KwaZulu-Natal, Durban, South Africa; 3 Department of Computer Science and Engineering, Texas A&M University, College Station, Texas, United States of America; 4 Department of Biochemistry and Biophysics, Texas A&M University, College Station, Texas, United States of America; 5 Department of Immunology and Infectious Disease, Harvard School of Public Health, Boston, Massachusetts, United States of America; 6 Department of Medicine, Groote Schuur Hospital and University of Cape Town, Cape Town, South Africa; University of Houston, United States of America

## Abstract

Bacterial cell wall components have been previously used as infection biomarkers detectable by antibodies. However, it is possible that the surface of the *Mycobacterium tuberculosis* (*M. tb*), the causative agent of tuberculosis (TB), also possesses molecules which might be non-antigenic. This makes the probing of biomarkers on the surface of *M. tb* cell wall difficult using antibodies. Here we demonstrate the use of phage display technology to identify peptides that bind to mycobacteria. We identified these clones using both random clone picking and high throughput sequencing. We demonstrate that random clone picking does not necessarily identify highly enriched clones. We further showed that the clone displaying the CPLHARLPC peptide which was identified by Illumina sequencing as the most enriched, binds better to mycobacteria than three clones selected by random picking. Using surface plasmon resonance, we showed that chemically synthesised CPLHARLPC peptide binds to a 15 KDa peptide from M.tb H37Rv whole cell lysates. These observations demonstrate that phage display technology combined with high-throughput sequencing is a powerful tool to identify peptides that can be used for investigating potential non-antigenic biomarkers for TB and other bacterial infections.

## Introduction

TB remains a significant problem worldwide, despite the widespread availability of effective antibiotics against drug sensitive *M. tb* strains. The World Health Organisation (WHO) estimates that in 2011, there were between 0.8 and 1.1 million deaths of HIV negative people globally, that resulted from TB [1]. Lack of rapid and accurate diagnostic tools limits the control of TB.

The absence of sensitive and specific TB detection reagents and a poor pipeline in biomarker identification significantly limits improvements in our ability to diagnose TB. One of the most desirable characteristics of a TB biomarker is its ability to differentiate patients with active disease from those with latent TB infection [2]. This may be best achieved by targeting a pathogen-associated biomarker as current immunological biomarkers are limited in their application: they are mainly used to detect latent infection and their specificity can be as low as 42% in high epidemic countries [3]. Thus far, the only available pathogen-associated tests that are used on sputum samples are smear microscopy [4], [5], culture [6], and nucleic acid amplification tests [7], [8]. In the case of extrapulmonary TB, or in paediatric and immunocompromised patients, where individuals would have difficulty producing a sputum sample, tests that probe for biomarkers that can be detected in samples other than sputum are critical. Currently, these include assays that detects lipoarabinomannan (LAM) [9], [10] in urine, the volatile organic compounds breath test [11], [12], and whole blood culture [13], [14]. However, these tests have varying limitations which include low sensitivity, low specificity or poor cost-effectiveness. Therefore, it is critical that new biomarkers are identified to improve diagnosis of TB.

We hypothesize that numerous cell wall associated components are shed by the mycobacterium during infection. These might possibly be detected in patient samples such as sputum, serum and urine, if their suitable probing reagents were available. Antibodies, which are the conventional reagents used for biomarker probing or pull-down are limited, because by definition they can only identify antigenic components. Thus, we employed phage display technology to identify peptides that can bind surface components of mycobacteria, regardless of their antigenicity.

Indeed, panning of phage display libraries has successfully identified peptides that bind intact bacteria [15] and viruses [16]. The technology involves the display of a random peptide sequence appended to a recombinant viral protein on the surface of a bacteriophage [17]. The typical selection, named biopanning, involves exposure of the unselected library to the target, and removal of unbound phages. The bound phages are then eluted and amplified by infection of host bacteria under selective pressure.

One of the challenging steps in the use of phage display technology is the identification of the most promising candidates at the end of the biopanning experiment. The random clone-picking method is traditionally used to sequence and identify displayed peptide clones that were enriched during biopanning. Depending on the sequence diversity at the end of the selection, this method may not necessarily identify the highly selected clones. However, high-throughput (HTP) sequencing has made possible the sequencing of millions of inserts allowing for a higher resolution of the selected pool of the displayed peptides [18], [19].

In this study, we used HTP sequencing to identify enriched peptide sequences from the biopanning experiment against *M. tb*. We employed a library that displays random 7-mer peptides (CX_7_C) at the tip of the pIII minor coat protein. The displayed peptides are flanked by two cysteine residues, which are oxidized during phage assembly to a disulfide bond, resulting in a loop constrained peptide. We initially used the traditional clone picking method to identify the enriched clones. This was followed by analyzing several rounds of selection through HTP sequencing. Surprisingly, we found that HTP sequencing not only revealed the dynamics of the selection but also identified the most abundant phage clone that was missed by the traditional clone picking method.

## Materials and Methods

### Bacterial strains and growth conditions

The bacterial strains used in the study were *M. tb* H37Rv, *M. tb* ΔleucineD and ΔpanthothenateCD double auxotroph (Δleu/Δpan), *M. smegmatis* mc^2^, *M. bovis* BCG and the *E. coli* ER2738 strain for phage amplification.

Mycobacteria were grown on Middlebrook 7H9 media (Sigma, St. Louis, MO) supplemented with the Middlebrook oleic albumin dextrose catalase (OADC) (bovine albumin fraction V 5 g/l, dextrose 2 g/l, catalase 0.004 g/l, oleic acid 0.05 g/l, sodium chloride 0.85 g/l) (Sigma, St. Louis, MO). The 7H9 media was further supplemented with both leucine (50 µg/ml) and pantothenate (24 µg/ml) as previously described [20]. For further use mycobacteria were centrifuged at 8000 rpm and resuspended in carbonate buffer (35 mM NaHCO_3_, 15 mM Na_2_CO3 [pH 9.8]). The *E. coli* ER2738 strain was grown in Luria-Bertani (LB) medium for amplification of phage. For the plating of the phage isopropyl β-D-thiogalactopyranoside (IPTG; 1 mM final concentration) and 5-bromo-4-chloro-3-indolyl-β-D-galactopyranoside (X-Gal; 60 mM final concentration) were added to LB agar plates before plating. All bacterial cultures were grown at 37°C with agitation.

### Immobilization of the target mycobacteria

Mycobacteria suspensions were prepared in carbonate buffer (35 mM NaHCO_3_, 15 mM Na_2_CO_3_ [pH 9.8]) and adjusted to an optical density of 1.0 at 660 nm, corresponding to approximately 10^8^ CFU per ml [15]. Maxisorp surface microtiter plate (Nunc, Roskilde, Denmark) wells were filled with 200 µl of bacterial suspension and incubated overnight at 4°C. Wells were blocked overnight at 4°C with gelatin (0.5%) supplemented supernatant of an *E. coli* strain ER2738 infected with the whole phage library [15].

### Selection of phage displayed peptides-biopanning

Selection of peptides from a CX_7_C library was carried out as previously described [21]. In the first round of panning, 10 µl of the library (∼2×10^11^ phages) was diluted to 100 µl with phosphate buffered saline supplemented with 0.1% Tween-20 (PBST) (0.01 M phosphate buffer, 0.0027 M potassium chloride and 0.137 M sodium chloride, pH 7.4, 0.1% tween) and incubated in bacterial-coated wells for 1 h at room temperature with gentle agitation. Nonbinding phages were then discarded as described by [21]. In brief, the wells were washed 25 times with PBST followed by four washes with the low pH elution buffer (0.2 M glycine-HCl, pH 2.2). Finally, the elution of the bound phage was carried out using 100 µl the low-pH elution buffer and sonicated in a sonicator water bath (50 kHz) for 10 minutes. The eluent was then neutralised with 15 µl of 1 M Tris-HCl, pH 9. In the following rounds of biopanning an average of 2×10^11^ plaque-forming units (PFU) was used. Five rounds of biopanning were performed. The first three rounds were targeted against *M. tb* (Δleu/Δpan), followed by a subtraction round against *M. smegmatis.* The fifth round of positive selection was against the targeted *M. tb* (Δleu/Δpan). After the final round of biopanning, single clones were picked and the random region sequenced using the −96M13gIII primer (5′-CCC TCA TAG TTA GCG TAA CG-3′). The unselected library, round 3, round 4 and round 5 phage pools; were also subjected to high throughput (HTP) Illumina sequencing.

### Phage Amplification

Eluates were amplified according to manufacturer's instructions (New England Biolabs, Beverly, Massachusetts). The phage eluates were used to infect *E. coli* ER2738 host cells. After 4.5 h of growth at 37°C, bacteria were removed by centrifugation and phages in the supernatant were precipitated by adding one-sixth volume of 20% polyethylene glycol-8000 and 2.5 M NaCl overnight at 4°C. The precipitate was resuspended in 100 µl of PBS, and amplified eluates were titered to determine phage concentration.

### Phage ELISA

Microtiter plate wells were coated with 100 µl of mycobacteria suspension, with an optical density of 1.0 at 660 nm in carbonate buffer (35 mM NaHCO_3_, 15 mM Na_2_CO_3_ [pH 9.8]), and incubated overnight at 4°C. Plates were blocked overnight at 4°C with 200 µl of the gelatin (0.5%)-supplemented supernatant of an *E. coli* strain ER2738 F′ culture infected with the whole phage library. A separate set of wells were blocked with blocking buffer without previous mycobacteria immobilization as negative controls (no target control). One hundred microliters of each selected amplified phage clone in PBS was transferred to coated wells. Plates were incubated for 1.5 h at room temperature. Wells were then washed six times with PBST. Horseradish peroxidise (HRP)-labelled mouse anti-M13 monoclonal antibody (GE Healthcare UK Ltd, Buckinghamshire, England) was diluted in PBS (1∶5,000). Two hundred microliters of the antibody was added per well and incubated for 1 h at room temperature. This was followed by washing the wells six times with PBST. One hundred and fifty microliters of substrate solution (2,2′-azino-di-[3-ethylbenzthiazoline sulfonate diammonium salt) (Thermo Scientific, Waltham, Massachusetts) was added and incubated for 30 min at 37°C. The reaction was stopped with 100 µl of 1% SDS. Absorbance was determined using a microtiter plate reader at the wavelength of 405 nm.

### Phage DNA preparation and sequencing

The amplification of the displayed peptides was performed with PCR using primers spanning the variable region in the gp3 phage coat protein (Table S1). The primers used for amplification contained homology required for annealing to the Illumina sequencing flowcell with the forward primer containing a five-nucleotide barcode to enable multiplexing. This enabled amplicons to be directly sequenced on an Illumina Genome Analyzer II as previously described [22]. The PCR reaction mix consisted of 0.5 µM of each primer, and 5 U of the GoTaq® DNA polymerase mix (Promega, Fitchburg, Winsconsin) in a 100 µl final volume. Whole phage PCR (denaturation at 95°C for 1 minute, annealing at 55°C for 2 minutes and extension at 72°C for 1 minute and 30 seconds) was performed as previously described [23]. Cycles varied from 10 to 25, and the number of reaction tubes varied from 2 to 5, according to the amount of amplicon available for each sample. Resulting amplicons (0.45–0.85 µg) were directly sequenced using the Illumina Genome Analyzer II. The nucleotide sequence of the amplified region of the gp3 gene was reconstructed by aligning and combining the two paired-end reads. The 36 bp variable region was extracted by trimming off constant bases and was translated into amino acid sequences of length 12 using the Illumina GA Pipeline software, which were then clustered for statistical analysis.

### Peptide Synthesis

Both peptides (Biotin-ACPLHARLPCG and its scrambled derivative Biotin-ACHLRPPLACG) were synthesised by GL Biochem (Shanghai, China), with the C-C disulphide bridge. The peptides were supplied as a powder with purity above 85%.

### Surface plasmon resonance (biosensor) analysis

A Biacore™ 3000 instrument (GE Healthcare UK Ltd, Buckinghamshire, England) was used. Instrument temperature was set to 25°C and HBS-N (10 mM Hepes and. 150 mM NaCl, pH 7.4) was used as running buffer. 50 µg/ml of streptavidin in sodium acetate buffer pH4.5, was immobilised by amine coupling on the CM5 sensor chip (GE Healthcare UK Ltd, Buckinghamshire, England). Immobilisation was performed at a flow rate of 10 µl/min for 7 min. The biotinylated peptides were captured using the previously immobilised streptavidin. A total of 60 µl of 100 µg/ml biotinylated-peptide in PBS (sample flow cell) was loaded onto the chip at the flow rate of 10 µl/min. No prior streptavidin or peptide was immobilised on the negative control flow cell. Binding of *M. tb* H37Rv whole cell lysate to the biotinylated peptide was then analysed by diluting the lysate in HBS-N buffer (10 mM Hepes and 150 mM NaCl, pH 7.4) and passing it over the chip at 10 µl/min. The *M. tb* H37Rv whole cell lysate binding was analysed at 100 and 500 µg/ml of total protein concentrations. While the unrelated bacteria whole cell lysates were analysed at 100 µg/ml of total protein concentration.

### Protease digestion of M. tuberculosis H37Rv lysate

*M. tuberculosis* H37Rv whole cell lysate was treated with 1 mg/ml Pronase E (Sigma, St. Louis, Missouri) for 2 hours. The protease digestion reaction was inactivated by heating at 90°C for 20 minutes. The negative control reaction was treated in a similar manner in the absence of Pronase E.

## Results

### Selection of phage displayed peptides binding to intact M. tb

**Figure 1:**
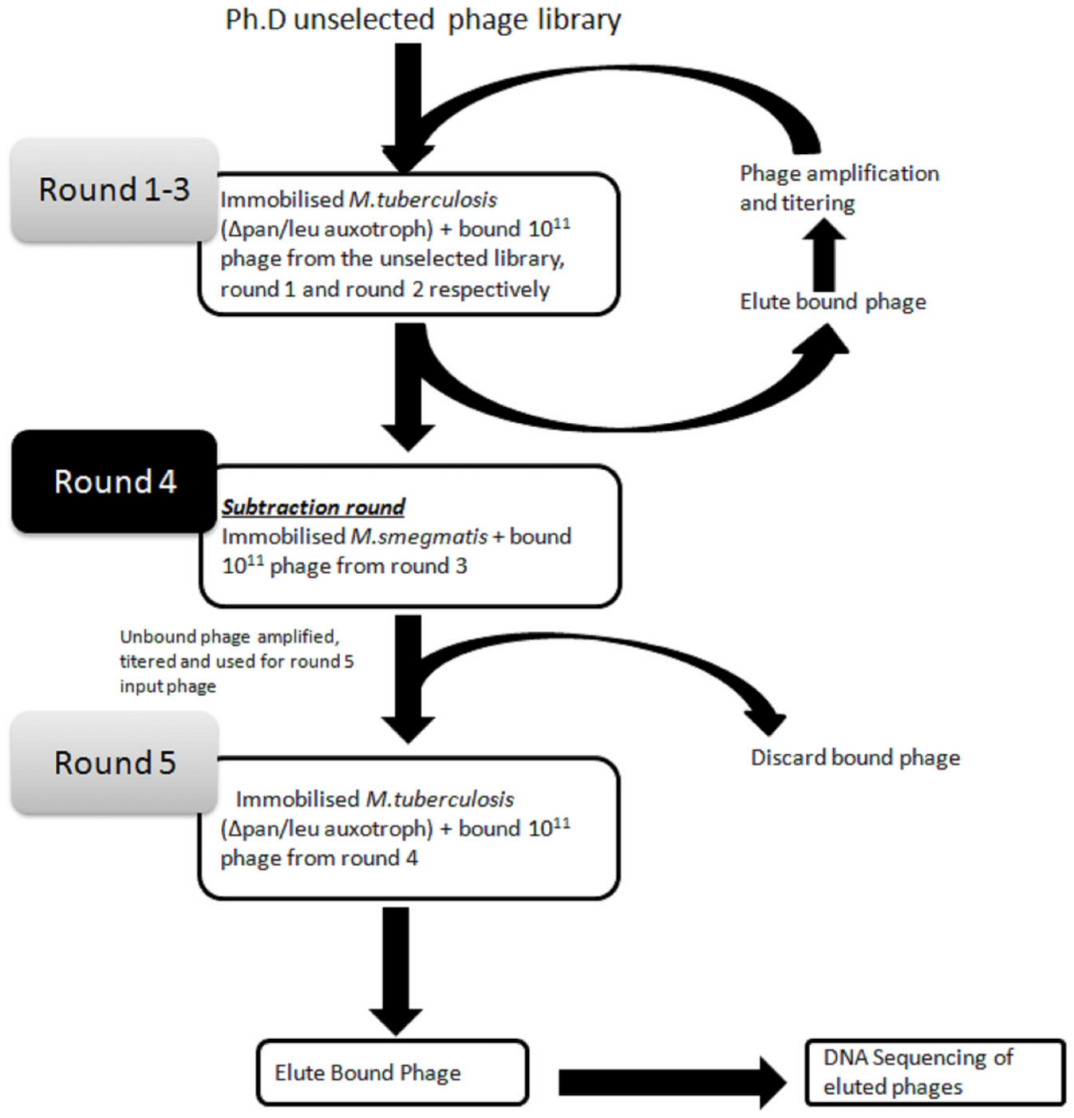
Schematic outline of the bio-panning procedure. Three rounds of biopanning were performed against the ΔleucineD and ΔpanthothenateCD double auxotroph (Δleu/Δpan) strain of *M. tb*. After the third round of panning, eluted phages were subjected to a subtraction round against *M. smegmatis* (round 4). Round 5 was performed against the target (Δleu/Δpan) *M. tb*. DNA from phages eluted at the end of the fifth round was sequenced and the corresponding peptide sequences were analysed.

**Figure 2:**
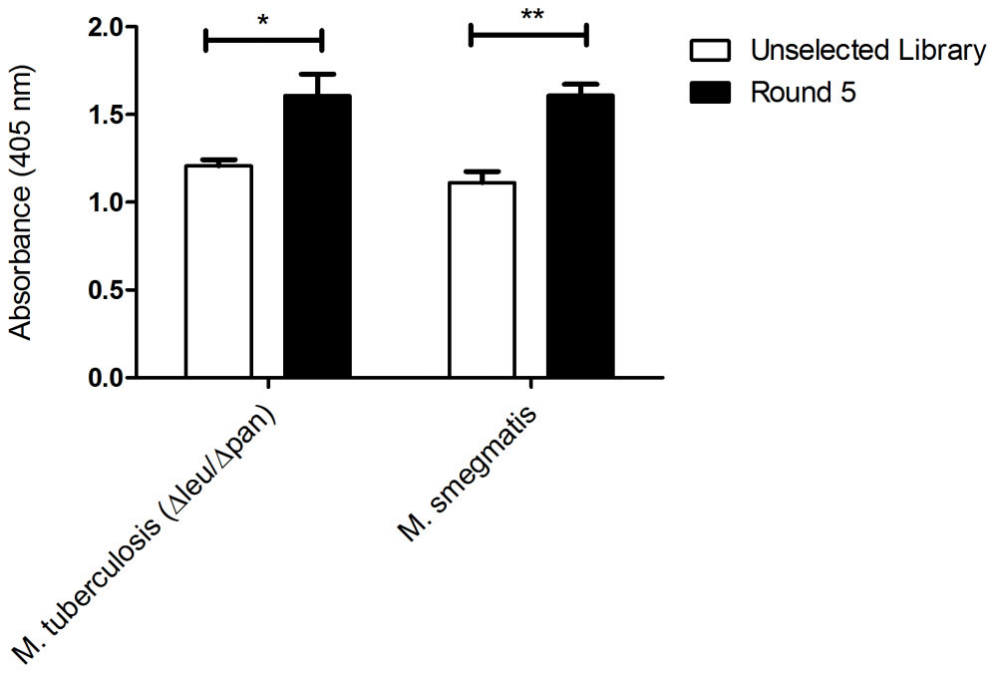
Binding of the phage eluates to mycobacteria species. Comparison of the binding signal of the unselected phage library to *M. tb* (Δleu/Δpan) and *M. smegmatis* to that of the phage population from the final round of biopanning (Round 5) to *the same species*.

In order to identify phage displayed peptides that could bind to intact *M.tb*, a constrained 7-mer (CX_7_C) phage library was used for panning on immobilised ΔleucineD and Δpanthothenate CD double auxotroph (Δleu/Δpan) strain of *M. tb*. This non-pathogenic *M. tb* strain was used as a model target because it is easy to manipulate, as it can be grown outside a biosafety level 3 laboratory. Three positive rounds of panning were performed against the targeted *M. tb* Δleu/Δpan strain (Figure 1). In order to remove peptides binding to cell wall components common to the mycobacterium genus, a subtraction round (round 4) was performed against *M. smegmatis*, which is a related mycobacterium. A final positive panning round (round 5) was performed to enrich for peptides that are specific to *M. tb* (Figure 1). The binding signal of the selected phage pool after five rounds of panning was significantly (p<0.05) higher when compared to that of the unselected library (Figure 2). This data suggests successful enrichment of clones that bind to mycobacteria.

### Characterisation of the enrichment process

**Figure 3:**
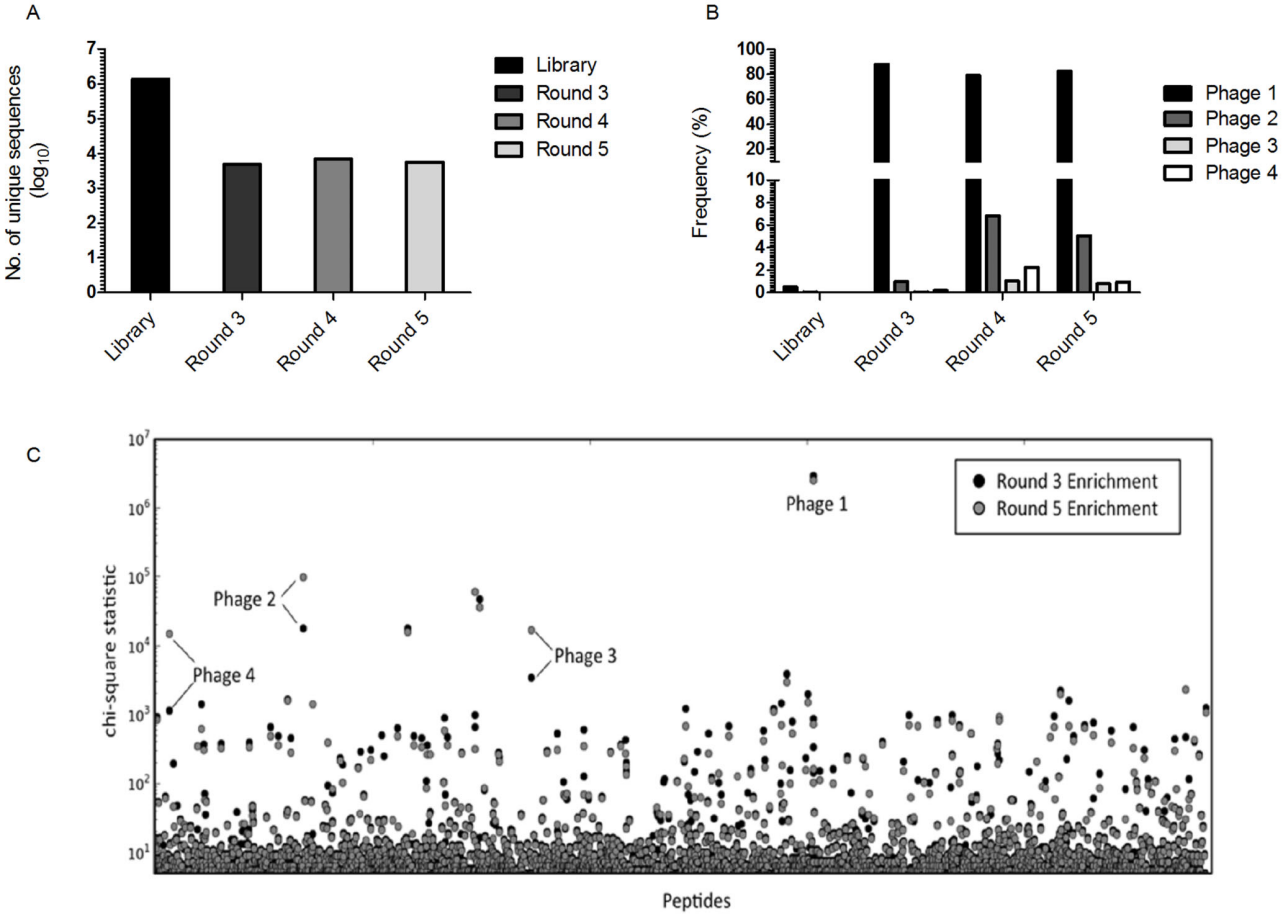
Sequence enrichment profiles after high-throughput sequencing of the phage displayed libraries using Illumina technology. (A) Number of unique peptides observed in the different rounds of biopanning (B) Frequency of the selected peptides at each round of biopanning (C) Manhattan plot showing peptide sequence enrichment (GWAS) results for round 3 and 5 of biopanning.

In order to evaluate the trend of enrichment during biopanning, we performed HTP sequencing on the library before selection and after three, four and five rounds of biopanning. We obtained approximately 1.5 million sequencing reads for each phage display selection round, representing 1.36×10^6^ unique peptides from the unselected library (Figure 3A). While this fell short of the theoretical complexity of the library, 1.23×10^9^ heptapeptides, it represented sufficient depth to measure the quantitative enrichment of relevant peptides. To confirm successful enrichment during selection, we characterized the reduction in diversity of the pool in the consecutive rounds of panning. The overall diversity decreased (Figure 3A) while the frequency of the highly enriched peptides increased (Figure 3B). To illustrate, the number of unique sequences decreased from 1,361,688 in the unselected library to 5665 after the final round of panning (Figure 3A). This suggests that there was enrichment during the panning experiments. Concurrently, the frequency of the most abundant peptide (corresponding to phage 1) increased from 0.48% to 81.15% in round three (Figure 3B), indicating that this peptide was highly selected for in as early as the third round of selection.

### Identification of highly selected phage clones

Ten plaques were selected using the traditional random cloning picking from the final round of biopanning, and were sequenced. Sequencing data of four of these plaques were ambiguous. Three unique sequences were obtained from the six remaining randomly selected plaques (Table 1). Two clones, phage 2 and phage 3, were represented more than once (Table 1). HTP sequencing, however, described a different quantitative landscape. We calculated the enrichment of every sequenced peptide by performing a Pearson Chi-squared test, comparing the selected pools to the input library (Figure 3C). While the proportion of multiple peptides increased in the selected libraries, a single phage clone displaying the peptide CPLHARLPC, dominated the selected libraries and was especially enriched during selection (p-value<10^−500^). Surprisingly, this clone was not identified using random clone picking (Table 1). Nonetheless there is still some degree of correlation between the peptide sequences that were identified by traditional clone picking and the top five sequences identified using high-throughput sequencing. That is, all three unique sequences identified during random clone picking were in the top five of the most abundant peptides identified by high-throughput sequencing. Moreover, since the most abundant peptide had a frequency of more than 80% after the first three rounds of selection, this means that with the current sequencing depth, further rounds of selection will less likely have lead to the identification of peptides that could not be found in the current available sequencing data.

**Table 1:** Summary of selected phage clones.

Phage Clone number	Percentage representation in the sequenced population				Phage displayed peptide sequence	SAROTUP: Target Unrelated Peptide scanner Results
	Random picking Method		HTP sequencing reads at round 5			
	Total number of sequences	Percentage representation of clone	Total number of sequence reads	Percentage representation of clone		
	n	%	n	%		
1^a^^,^ †	6	0	1 655 954	82.49	CPLHARLPC	Anti-influenza A H1N1 monoclonal antibody IV.C102 and SIV sera from patients (Zhang *et al*., 2011)
2^a^^,^ b	6	16.67	1 655 954	0.81	CHYDGARAC	None found
3^a^^,^ b	6	33.33	1 655 954	0.92	CDHGYLPSC	None found
3^a^^,^ b	6	50.00	1 655 954	5.05	CFDTRSLVC	None found

a Clones identified in the top ten highly enriched sequenced by HTP sequencing.

b Clones identified through random sequencing.

† The highest enriched clone at round 5, as identified by HTP sequencing.

To establish whether the selected peptides were not binding to non-targeted substrates and other components used during the biopanning process like BSA, we used the web-based server called Scanner and Reporter Of Target-Unrelated Peptides (SAROTUP), which can identify non target specific peptides [24]. None of the four sequences selected were identified as nonspecific binders to the commons reagents used during selection. However, phage 1 has recently been isolated and characterised as binding to the IV.C102 H1N1 monoclonal antibody and the swine-origin influenza virus A sera [25] (Table 1).

### Binding characterisation of the selected recombinant phages to *M. tb*

**Figure 4:**
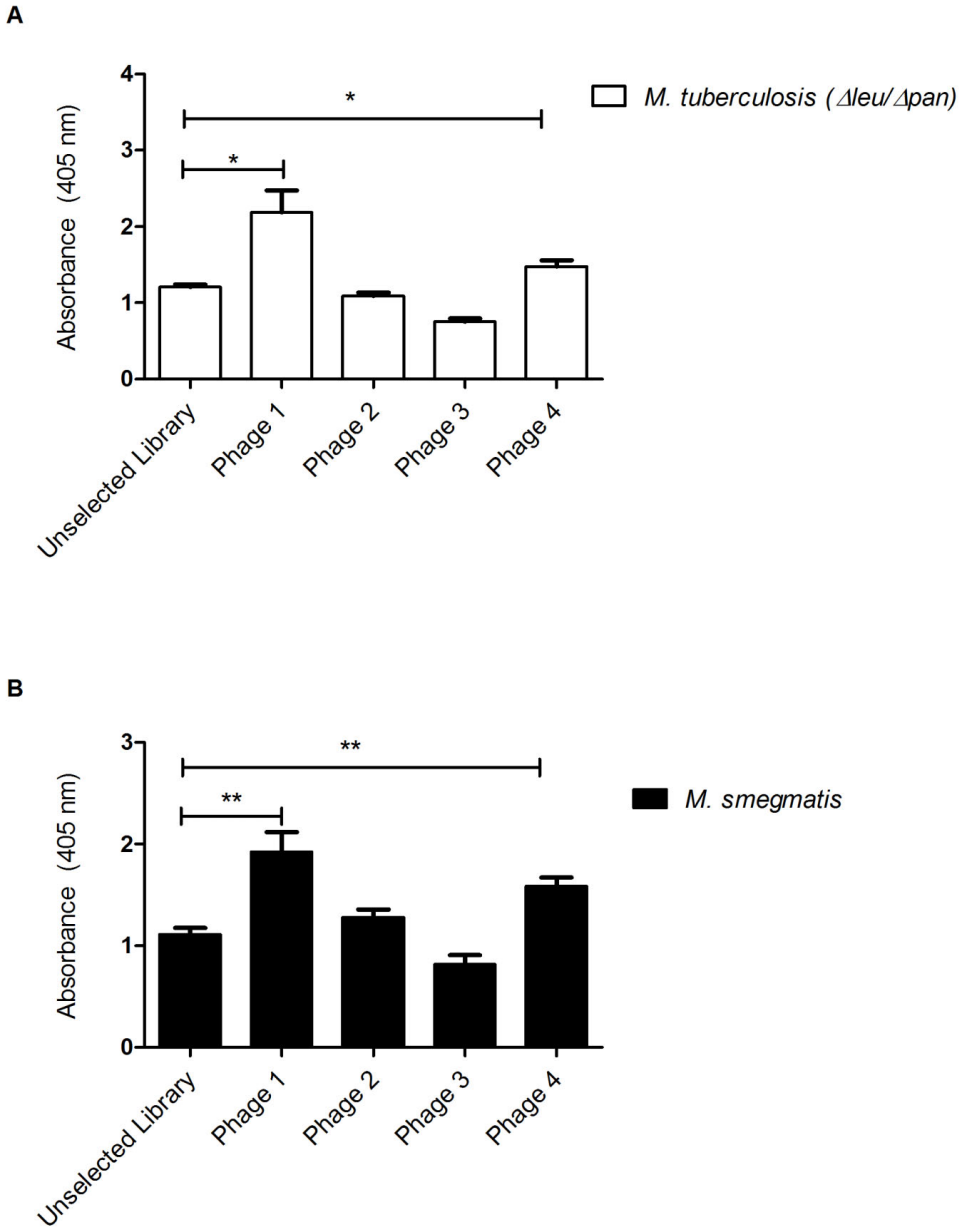
Binding of the selected phage clones to mycobacteria species. (A) *M. tb* (Δleu/Δpan), (B) *M. smegmatis*. A two-tailed, unpaired t-test was used to analyse significance (**p<0.01 and *p<0.05). Error bars represent standard deviations of the arithmetic means of the normalised optical densities at 405 nm performed in triplicates.

Binding of the selected phage clones was investigated on immobilised *M. tb* (Δleu/Δpan) strain (Figure 4B). We further evaluated the effectiveness of the subtraction round, by comparing the binding of the selected phages to *M. tb* (Δleu/Δpan) strain to that of *M. smegmatis* (Figure 4C) which was targeted during the subtraction round. For each amplified clone, 10^11^ PFU were used in the phage ELISA. Our results showed that phage 1 which was identified using HTP sequencing and one out of the three phages identified by random cloning, phage 4, had significantly (p<0.05) higher binding to *M. tb* as compared to the unselected library (Figure 3B). Interestingly, phage 1 and phage 4, in addition to significantly binding to intact *M. tb*, also showed significant (p<0.01) binding to *M. smegmatis* (Figure 4C). However, phage 2 and 3 which were respectively identified using random clone picking showed no significant binding to both *M. tb* and *M. smegmatis* as compared to the unselected library (Figure 4B–C).

### Binding characterisation of the highly enriched, HTP sequencing identified Phage 1

**Figure 5:**
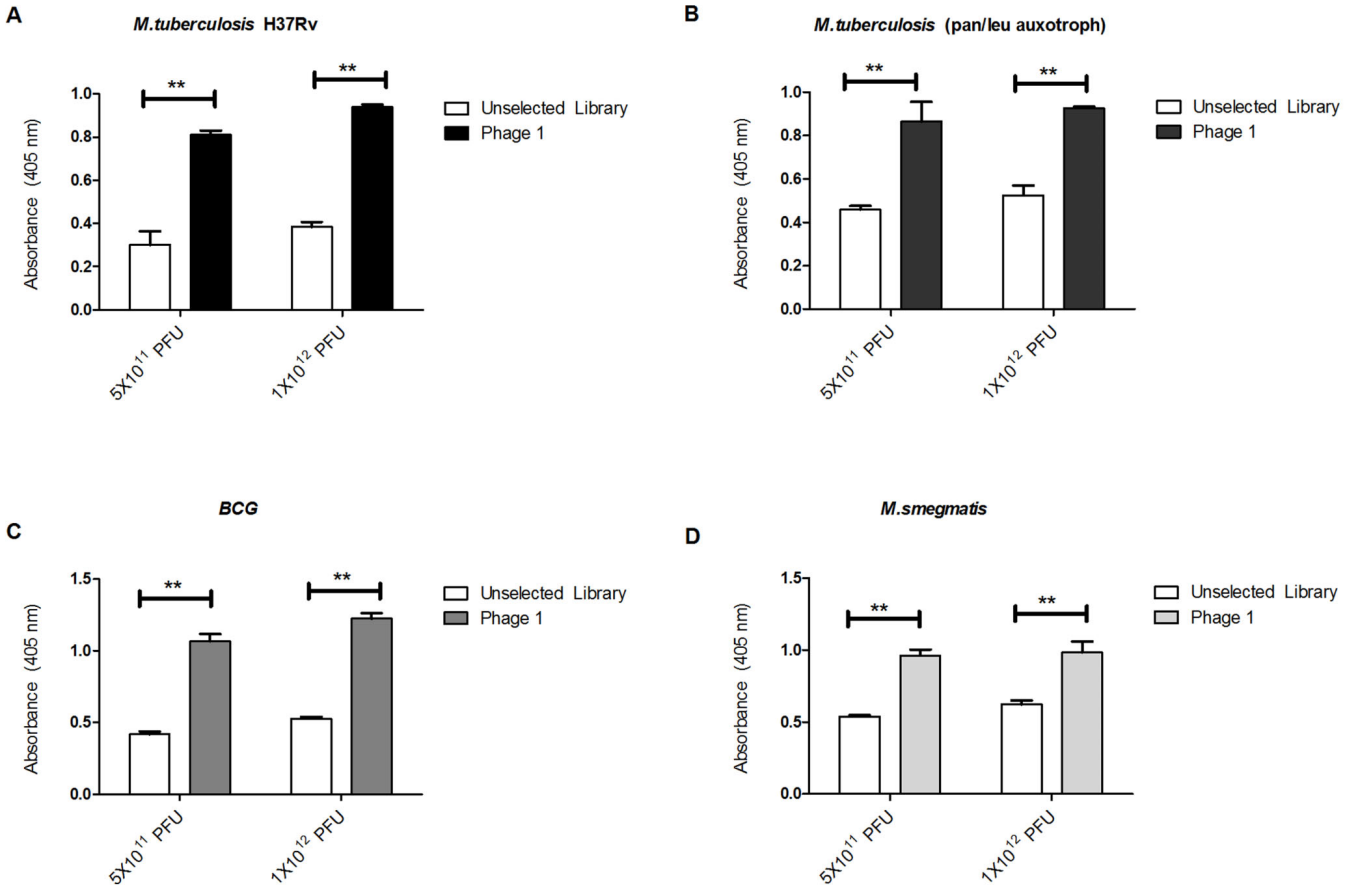
Characterisation of the Phage 1 clone identified using HTP Illumina sequencing. Phage 1 was amplified and 5×10^11^, 1×10^12^phages (x-axis) were used for a plate binding assay with (A) *M. tb* H37Rv, (B) (Δleu/Δpan) *M. tb*, (C) *BCG* and (D) *M. smegmatis* as solid-phase antigens. The unselected library was used as a control. A two-tailed, unpaired t-test was used to analyse significance (**p<0.01). Error bars represent standard deviations of the arithmetic means of the normalised optical densities at 405 nm performed in triplicates.

The highly enriched recombinant phage 1 was further investigated on its ability to bind the pathogenic strain of *M. tb* H37Rv, using ELISA. To evaluate whether the selected phage could discriminate between bacteria from the same genus of mycobacterium, we further characterised its binding specificity by comparing its binding to *M. tb* and to other mycobacterium species namely, *M. smegmatis*, *M. bovis* BCG and the *M. tb* (Δleu/Δpan) strain. Our results show that phage 1 consistently bound to all the three mycobacterium species tested, which included two strains of *M. tb*, with a binding signal that was at least twice as high when compared to the phage library which was used as a control (Figure 5A–D). These results were consistent when tested using two different phage inputs of 5×10^11^ and 1×10^12^ PFU. This phage showed no significant difference in binding the pathogenic strain *M. tb* H37Rv, *M. tb* (Δleu/Δpan) or *M. smegmatis*. Notably, there was a significantly (p<0.01) higher binding signal to BCG as compared to *M. tb* H37Rv.

### Binding of the synthetic peptide displayed by phage 1 to *M. tb* H37Rv lysate

**Figure 6:**
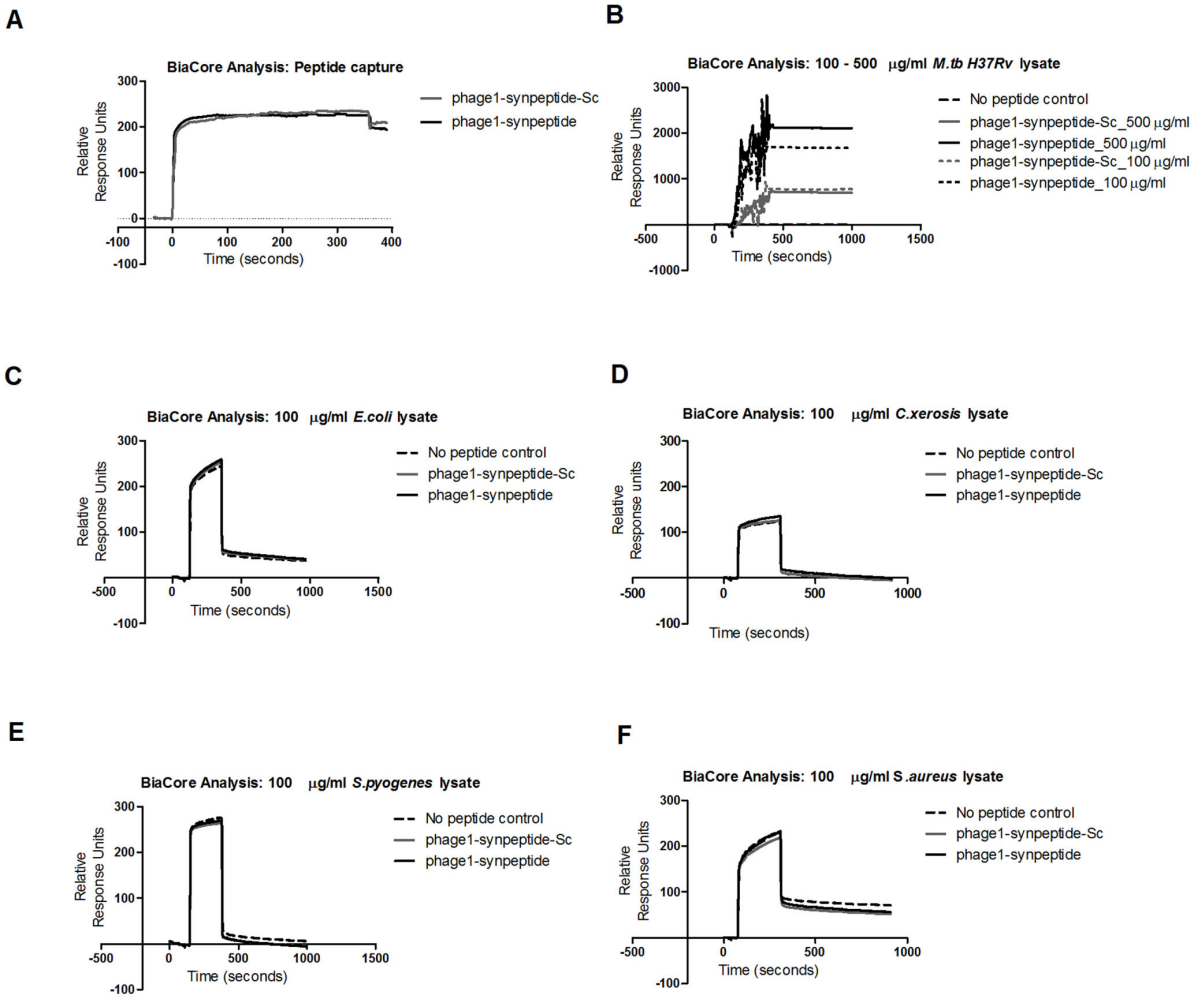
Biacore sensograms showing the association of bacteria whole cell lysates with immobilized synthetic peptides. (A) Phage1 synthetic peptide (phage1-synpeptide) and scrambled peptide (phage1-synpeptide-Sc) were captured with the covalently bound streptavidin on the CM5 chip. (B) Whole cell lysates of *M. tb* H37Rv with a total protein concentration of 100 µg/ml and 500 µg/ml were injected over the immobilised peptides. To evaluated specificity whole cell lysates with a total protein concentration of 100 µg/ml from unrelated bacteria, (C) *Escherichia coli* ER2738 (D) *Corynebacterium xerosis* (E) *Streptococcus pyogenes* (F) *Staphylococcus aureus*, were injected over the immobilised peptides. Changes in surface plasmon resonance were monitored in real time and are shown in response units.

In order to test whether the highly enriched displayed peptide can bind its ligand when it is not displayed by the carrier phage, the peptide CPLHARLPC (phage1-synpeptide) and its scrambled derivative CHLRPPLAC (phage1-synpeptide-Sc) were synthesised with a biotinylation modification. The binding of the synthesised peptides to the whole cell lysate from *M. tb* H37Rv was evaluated using the biacore SPR technology. Biotinylated peptides were immobilized on a streptavidin sensor chip (Figure 6A) and whole cell lysates were injected at different concentrations of total protein. When evaluating the binding of the synthesized peptides to different concentrations of *M. tb* whole cell lysate, we found that there was a 25% increase in binding signal of phage1-synpeptide when the total protein concentration of the lysate was increased by 5 fold from 100 µg/ml to 500 µg/ml. In contrast, there was no notable difference in the binding signal of the scrambled derivative between the two concentrations (Figure 6B). This data suggests that, Phage1-synpeptide binds more strongly to H37Rv whole cell lysate than its scrambled derivative. To further evaluate specificity of the selected peptides, we measured their binding signal to whole cell lysates from unrelated bacteria. All bacteria strains tested which included both Gram negative and Gram positive bacteria that are potential upper respiratory pathogens and an *E. coli* strain used for the amplification of phage during biopanning, had binding signal similar to that of the negative control, which had no prior peptide immobilisation (Figure 6C–F). This data is indicative of the specificity of the selected peptide to binding mycobacteria.

### Characterisation of the mycobacteria cell wall associated binding partner for Phage1 synthetic peptide (phage1-synpeptide)

**Figure 7:**
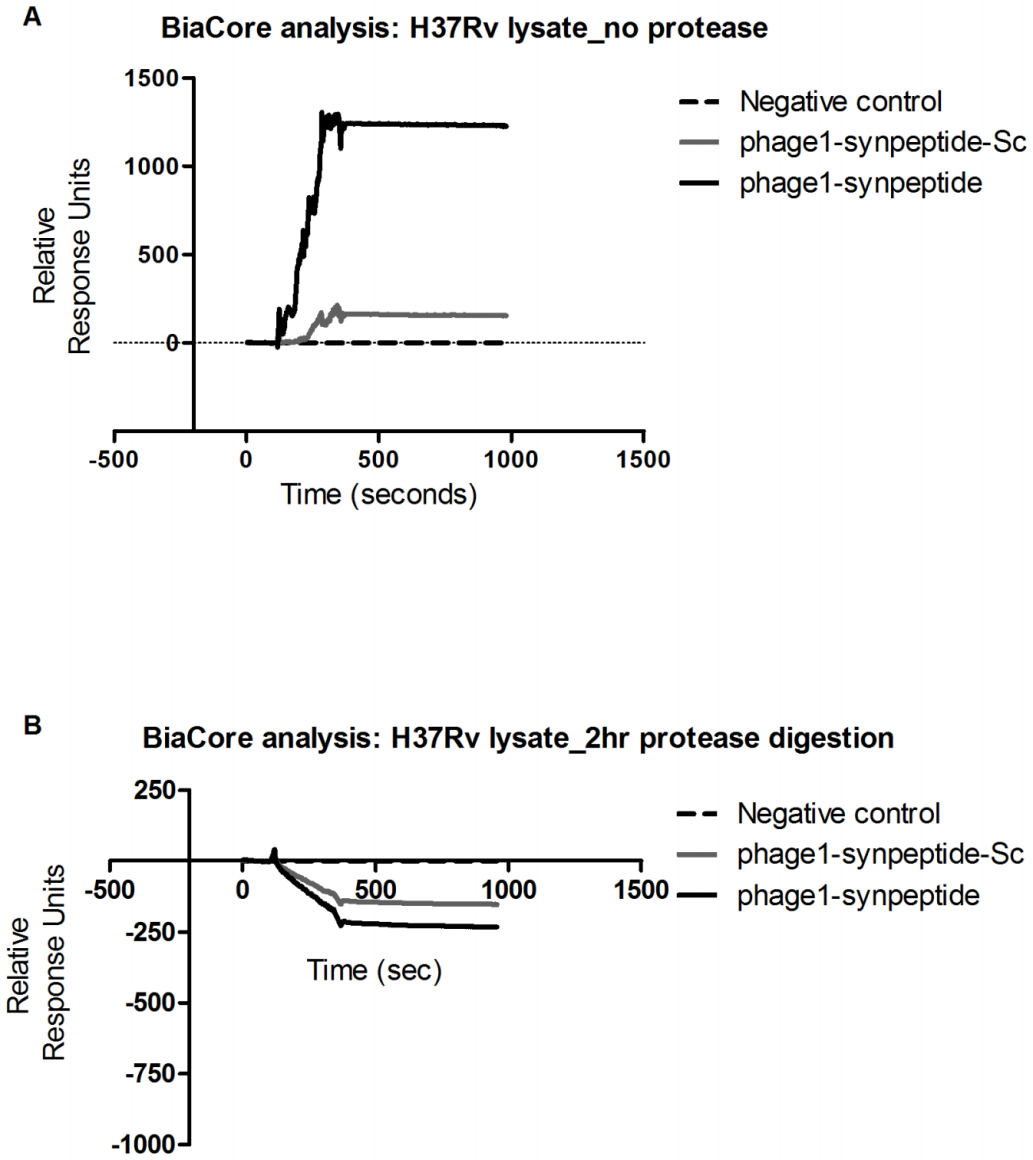
Biacore sensograms showing the association of protease digested *M. tb* lysate with immobilized synthetic peptides. Biotinylated phage1 synthetic peptide (phage1-synpeptide) and biotinylated phage 1 scrambled peptide (phage1-synpeptide-Sc) were captured with covalently bound streptavidin on a CM5 chip. Protease-digested whole cell lysate of *M. tuberculosis* H37Rv with a total protein concentration of 100 µg/ml was injected over the immobilised peptides. (B) shows tha control experiment using the undigested *M. tuberculosis* H37Rv whole cell lysate. Changes in surface plasmon resonance were monitored in real time and are shown in response units.

To determine if the mycobacteria target of phage 1 displayed peptide is a protein, we tested its binding to protease-treated *M. tuberculosis* whole cell lysate. Our results showed that the binding of phage1-synpeptide to *M. tuberculosis* whole cell lysate is abrogated after the lysate has been incubated for 2 hrs with Protenase E (Figure 7). This data suggests that phage1-synpeptide binding partner is likely to be of a peptide or protein nature.

**Figure 8:**
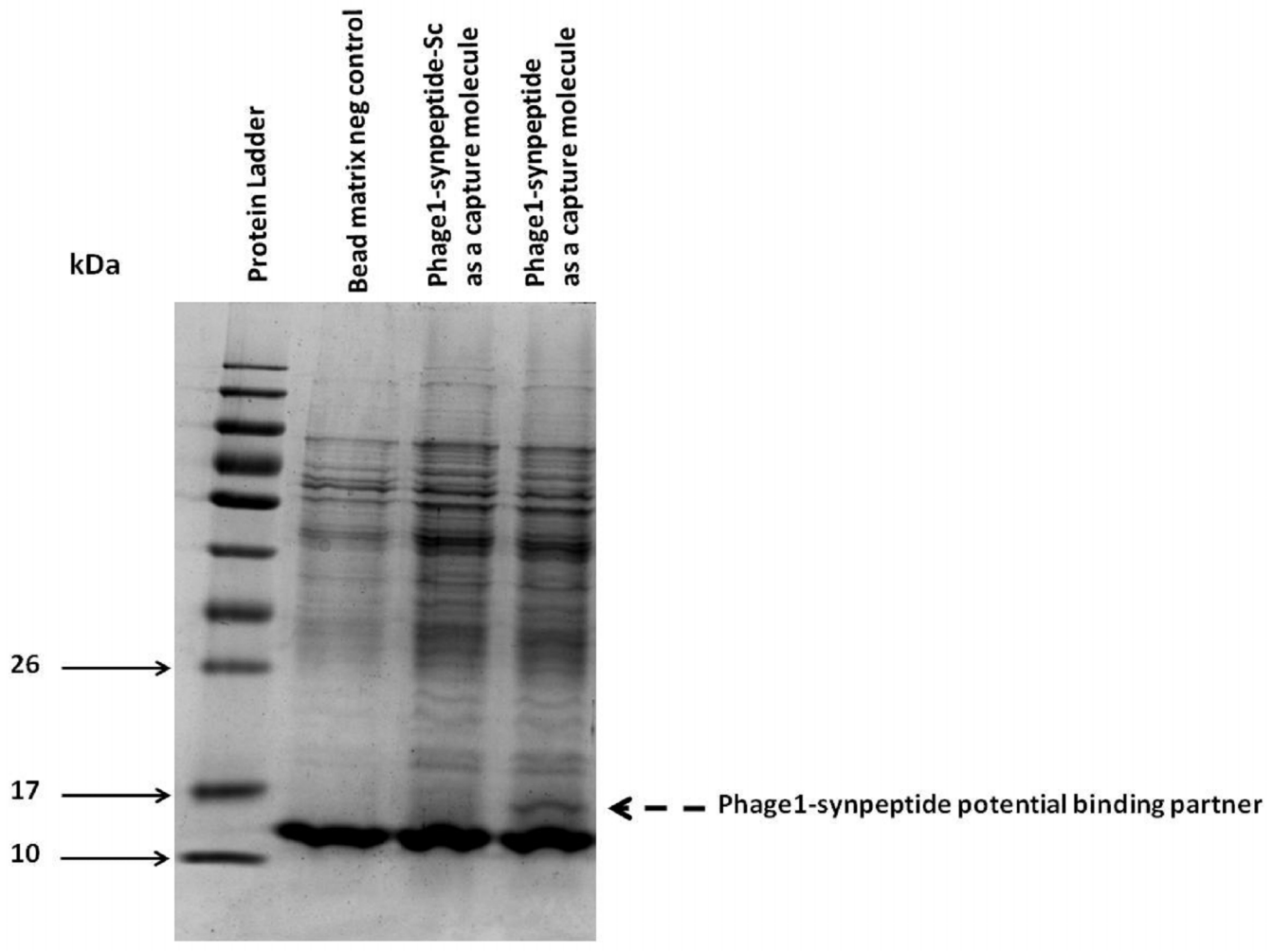
SDS–PAGE showing proteins pulled down from *M. tb* H37Rv whole cell lysate. Coomassie stained 12% SDS–polyacrylamide electrophoresis gel of proteins obtained from pull down experiments. Lane 1: molecular weight protein ladder. Lane 2: proteins pulled down from *M. tuberculosis* H37Rv whole cell lysate by the streptavidin beads in the absence of phage1-synpeptide. Lanes 3: proteins pulled down from *M. tuberculosis* H37Rv whole cell lysate by the streptavidin beads that had been pre-incubated with 0.5 mg of biotinylated phage1-synpeptide-Sc. Lane 4: proteins pulled down from *M. tuberculosis* H37Rv whole cell lysate by the streptavidin beads that had been pre-incubated with 0.5 mg of biotinylated phage1-synpeptide. The dotted arrow indicates the possible bio-phage1-synpeptide binding protein from *M. tuberculosis* H37Rv.

In order to validate that mycobacteria protein interacts with the phage 1 displayed peptide, we performed a pull down assay from *M. tuberculosis* H37Rv whole cell lysate using phage1-synpeptide as a capture peptide. The scrambled derivative, phage1-synpeptide-Sc, and the streptavidin beads without prior immobilisation of the phage 1 peptide were used as negative controls. A protein of approximately 15 kDa in size was pulled down from *M. tuberculosis* whole cell lysate by phage1-synpeptide and was not detectable on the PAGE gel using Coomassie staining on both negative control experiments (Figure 8). The absence of the pulled down peptide when phage1-synpeptide-Sc (Figure 8) was used as capture molecule, is indicative of the specific nature of the interaction between the phage 1 displayed peptide and its mycobacteria binding partner.

## Discussion

In this study, we applied phage display technology with the aim of searching for peptide ligands that bind to *M. tb*, and can be later developed to probe for potential biomarkers in patients' clinical samples. We evaluated four phage clones that were identified from the panning of a loop-constrained heptapeptide (CX_7_C) library against *M. tb*. Both HTP sequencing and random clone picking revealed multiple peptides that were enriched during selection. However, only with HTP sequencing were we able to calculate quantitative measures of enrichment, which allowed us to compare and to rank our multiple hits. We found that our most enriched peptide which was identified only by HTP sequencing had the highest binding signal to Mtb when compared to all of the randomly selected clones and the unselected library. A single phage clone, phage 1, displaying the peptide CPLHARLPC, was significantly enriched during the selection. Interestingly, the traditional random clone picking method failed to identify this clone, despite the fact that HTP sequencing showed that this clone represented over 80% of the sequenced population after the first three rounds of selection (Figure 3B). This result is in agreement with earlier findings showing that when comparing HTP sequencing to the traditional clone picking method, HTP sequencing accelerates the discovery of specific binders [19]. This earlier independent study supports our finding by demonstrating the high correlation between abundances in the first round and subsequent rounds of selection, clearly showing that HTP sequencing could identify the highly enriched clones without the need of additional selection rounds [19]. The higher resolution of the selection pools enabled by HTP sequencing also allowed us to characterize the enrichment process. Firstly, by demonstrating the reduction in the number of unique peptides during biopanning (Figure 3A), which is indicative of the enrichment of a subset of phages. Secondly, by revealing that phage 1 was significantly enriched to 80% of the sequenced population as early as round 3 (Figure 3B). Attaining this kind of quantitative data would not be possible using random clone picking, since this method is limited by the number of clones that can be analyzed. This means that it will be improbable to achieve the sequencing depth similar to that of HTP sequencing, making it more difficult to evaluate the degree of enrichment of the selected clones.

When we further characterized the binding specificity of phage 1 to pathogenic *M. tb* H37Rv, it was interesting to note that there was no significant difference in the binding to all mycobacterium species tested, with the exception of BCG which had a significantly (p<0.01) higher binding signal. This data suggests that the phage 1 binds to a molecule conserved across the three mycobacterium species.

A phage displaying the same peptide, CPLHARLPC, has recently been isolated and characterized as binding to the IV.C102 H1N1 monoclonal antibody and the swine-origin influenza virus A sera [25]. This monoclonal antibody has previously been demonstrated to bind to the type A H1N1 influenza strain epitopic peptide localized in residues 207–225 of the hemagglutinin HA1 subunit [26]. However, there is no sequence similarity between the IV.C102 monoclonal antibody epitope (AIYHTENAYVSVVSSHYNR) on the hemagglutinin protein and the peptide displayed by phage 1. Luchesse and colleagues (2009) further characterized that peptide AIYHTENA is the minimal determinant epitopic region required for IV.C102 binding [27], which only has two amino acids in common, histidine and alanine, to the phage 1 displayed peptide. Nevertheless, the phage 1 displayed peptide contains five hydrophobic amino acids (out of nine), and the minimal epitopic peptide of the IV.C102 antibody also includes four hydrophobic amino acids (out of the eight). This may suggest that the interaction of this peptide with either the IV.C102 H1N1 monoclonal antibody or mycobacteria is most likely via hydrophobic interactions.

There are reagent similarities, like the plastic polystyrene microtiter wells, between the biopanning experiments and an ELISA. It is possible that these similarities could contribute falsely to apparent binding as previously discussed by others [28]. This necessitates the use of a different method that does not include these materials, to further validate intended target binding. Indeed, when the peptide displayed by phage 1 was evaluated on surface plasmon resonance technology, the results showed that this peptide is able to associate with the *M. tb* H37Rv whole cell lysate while its scrambled counterpart exhibited minimal binding (Figure 6B). The diminished binding of this peptide when it is scrambled shows that the phage1-synthetic peptide sequence is important for its specific interaction with mycobacteria. Furthermore, this peptide showed no binding (Figure 6C–F) to unrelated bacteria that were tested which is indicative of specificity to mycobacteria.

Mycobacteria have multiple possible binding partners for the phage 1 displayed peptide. These potential ligands vary in their nature, ranging from cell wall proteins, glycans and free lipids [29]. In this work, we have demonstrated that the highly enriched phage 1 peptide binds to a mycobacterial protein of approximately 15 kDa in size. However, the identification of the target protein requires further validation.

In conclusion, our findings show that phage display combined with HTP sequencing is a useful tool for the identification of specific peptides to mycobacteria. Our results also indicate that peptide CPLHARLPC is a good candidate to probe for a potential biomarker for TB infection. However, the lack of specific mycobacterium strain markers remains a limiting factor in TB biomarker development. Notwithstanding, this is a proof-of-concept study showing that this approach could be used to identify additional peptides with better specificity for *M. tb*.

## Supporting Information

Table S1 **Primer sequences used in PCR amplification for generating Illumina sequencing templates.**
(DOCX)
