# Supplementary material for: High-Throughput Sequencing Enhanced Phage Display Identifies Peptides That Bind Mycobacteria
Source: PLoS One. 2013 Nov 12;8(11):e77844. doi: 10.1371/journal.pone.0077844 (PMC3827053; doi:10.1371/journal.pone.0077844)
Supplement: Table S1 — Primer sequences used in PCR amplification for generating Illumina sequencing templates. (DOCX) [file pone.0077844.s001.docx]

Title: High-throughput sequencing enhanced phage display identifies peptides that bind mycobacteria

**Supplementary online data**

**Nqobile A. C. Ngubane^1,2^, Lionel Gresh^1^, Thomas R. Ioerger^3^, James C. Sacchettini^4^, Yanjia J. Zhang^5^, Eric J. Rubin^5^, Alexander Pym^2^ , and Makobetsa Khati^1, 6*^**

**1**Emerging Health Technologies Platform, Council for Scientific and Industrial Research, Biosciences Unit, Pretoria, Gauteng, South Africa, **2**KwaZulu-Natal Research Institute for Tuberculosis and Human Immunodeficiency Virus, Nelson R. Mandela School of Medicine, University of KwaZulu-Natal, Durban, South Africa, **3**Department of Computer Science and Engineering, Texas A&M University, College Station, Texas, United States of America, **4**Department of Biochemistry and Biophysics, Texas A&M University, College Station, Texas, United States of America, **5**Department of Immunology and Infectious Disease, Harvard School of Public Health, Boston, Massachusetts, United States of America, **6**Department of Medicine, Groote Schuur Hospital and University of Cape Town, Cape Town, South Africa

^*^E-mail address: [Mkhati@csir.co.za](mailto:Mkhati@csir.co.za)

**Online Supplementary data**

**Table S1: Primer sequences used in PCR amplification for generating Illumina sequencing templates.**

| **Phage population used as a PCR template** | **Primer sequence** |
| --- | --- |
| Unselected Library (Forward primer) | F:5'-CAAGCAGAAGACGGCATACGAGAT***ATTGGC***GTGACTGGAGTT  CAGACGTGTGCTCTTCCGATCTTTCGGCCGAACCTCCACC-3' |
| Round 3 (Forward primer) | F:5'-CAAGCAGAAGACGGCATACGAGAT***GATCTG***GTGACTGGAGTT  CAGACGTGTGCTCTTCCGATCTTTCGGCCGAACCTCCACC-3' |
| Round 4 (Forward primer) | F:5'-CAAGCAGAAGACGGCATACGAGAT***TCAAGT***GTGACTGGAGTT  CAGACGTGTGCTCTTCCGATCTTTCGGCCGAACCTCCACC-3' |
| Round 5 (Forward primer) | F:5'-CAAGCAGAAGACGGCATACGAGAT***CTGATCC***GTGACTGGAGTT  CAGACGTGTGCTCTTCCGATCTTTCGGCCGAACCTCCACC-3' |
| Reverse primer for all phage populations | R: 5'-AATGATACGGCGACCACCGAGATCTACACTCTTTCCCTACAC  GACGCTCTTCCGATCTTTTAGTGGTACCTTTCTATTCTCACTCT-3' |

^a^The reverse primer was the same for all PCR amplification reactions. The multiplex sequencing tag is bolded and underlined in all forward primers.
